# Prevalence of perinatal depression in Ethiopia: An umbrella review of systematic review and meta-analysis studies

**DOI:** 10.1371/journal.pone.0347570

**Published:** 2026-04-27

**Authors:** Mogesie Necho, Maregu Shegaw, Tamirat Anbesaw, Nahom Birru, Getinet Ayano, Mekonnen Tsehay, Wondale Getnet, Solomon Yimer, Asmare Belete, Yosef Zenebe, Zelalem Birhan, Chalachew Tiruneh

**Affiliations:** 1 Flinders University Adelaide, South Australia; 2 Department of Psychiatry, College of Medicine and Health Science, Wollo University, Ethiopia; 3 School of Population Health, Curtin University, Perth, Australia; 4 Departments of Psychiatry, College of Medicine and Health Science, University of Gondar, Ethiopia; 5 College of Medicine and Health Science, Flinders University, South Australia; 6 Department of Medical Anatomy, College of Medicine and Health Science, Injibara University, Ethiopia; National Institute of Mental Health and Neurosciences: National Institute of Mental Health and Neuro Sciences, INDIA

## Abstract

**Background:**

Perinatal depression is a significant public health concern that affects women during pregnancy and the postpartum period. Despite being acknowledged globally, the burden of perinatal depression is particularly profound in low and middle-income countries, such as Ethiopia. This umbrella review is therefore intended to systematically consolidate findings on perinatal depression among Ethiopian women to better understand its prevalence, thereby highlighting the gaps in current research and informing future interventions.

**Methods:**

This umbrella review used the PRIOR checklist for the reviews of systematic review and meta-analytic studies. The review protocol has been registered on PROSPERO: CRD42023495174. PubMed, EMBASE, and PsycINFO databases were searched for the presence of systematic review and meta-analysis studies. The quality of included articles has been evaluated with a measurement tool to assess systematic review and meta-analysis studies (AMSTAR). A novel graphic approach with an estimated corrected covered area (CCA) has been used to determine the degree of overlap of primary studies in the systematic review and meta-analysis studies. The weighted random effect model was used during the meta-analysis.

**Result:**

A total of 28 unique primary studies and 8 systematic reviews, and meta-analysis studies with 15,592 participants were included in this umbrella review. The pooled prevalence of perinatal depressive symptoms in the included systematic review and meta-analysis studies ranges from 20.1% to 25.8%. The pooled umbrella prevalence of perinatal depressive symptoms among women in Ethiopia was 22.49% (95 CI%:21.38, 23.59). The pooled umbrella analysis revealed that the antenatal and postnatal depressive symptoms were 22.76% (95% CI: 19.9, 25.62) and 21.75% (95% CI: 21.03, 22.48), respectively. In addition, the pooled prevalence of perinatal depression in studies that included 10 or below primary studies is 22.86% (95%CI:20.39, 25.33), and in those that included below 10 primary studies, it was 22.10% (95%CI: 21.55, 22.65). The novel graphic presentation depicted a very high degree of overlap of primary studies in the included systematic reviews and meta-analysis studies; corrected covered area (CCA) of 25.5%. Four of the included studies (fifty percent) had high methodological quality, and the remaining four relied on a moderate quality range.

**Conclusion:**

The pooled overall, antenatal, and postnatal prevalence of depression symptoms was high in Ethiopia, with no significant difference during the antenatal and postnatal period. An improved understanding of perinatal depression will therefore guide policymakers and health practitioners in developing targeted strategies to alleviate this mental health challenge.

## 1. Introduction

Perinatal depression (PND) is a pressing public health issue in Ethiopia, with significant implications for maternal and child health [1–9]. PND, encompassing both antenatal (during pregnancy) and postpartum periods, significantly impacts maternal and offspring well-being [10–13]. National estimates illustrated that the prevalence of antenatal depression around the world was 20.7%, with variances among high-income countries (9%) and low-income countries (19%) [14,15]. Meta-analyses of maternal depression in high-income countries reported a prevalence of 11% during pregnancy and 13% in the postnatal period [16]. Another meta-analysis of maternal depression with data from 21 countries estimated a prevalence of 9.76% during pregnancy and 8.75% during the first postnatal year [17].

Studies indicated that the prevalence of perinatal depression in Ethiopia ranges from 10% to over 30%, highlighting a considerable burden on women’s health [1–9]. Different systematic review and meta-analysis studies on perinatal mental health in Ethiopia reported the prevalence of antenatal depressive symptoms within a range of 21.28% to 24.2% [1,9], and postnatal depressive symptoms between 20.1% to 22.28% [6,18–21].

Despite its prevalence, PND remains under-recognized and undertreated due to a lack of awareness, cultural stigma, and limited access to mental health resources [22–25]. The multifactorial nature of PND, influenced by socio-economic, cultural, and healthcare system factors, complicates its identification and management in the Ethiopian context [1–9,26–30]. Traditional beliefs surrounding mental health often hinder open discussions and proper interventions, resulting in a gap in care and support for affected women [22–25,31,32] Furthermore, healthcare providers may lack the necessary training and tools to effectively screen and treat perinatal depression, exacerbating the issue.

Despite the growing body of literature on perinatal depression globally, there is a notable lack of comprehensive reviews that synthesize existing research specific to Ethiopia. In addition, many individual and systematic review studies in Ethiopia have explored various magnitudes of perinatal depression [1–9,22,23,26–30], the sheer volume of fragmented meta-analyses and methodological controversies hinder a cohesive understanding of its burden. Additionally, the credibility of evidence requires rigorous evaluation of the magnitude of perinatal depression [33]. This knowledge gap impedes the development of targeted interventions and policies to address PND effectively. An umbrella review is therefore essential to consolidate findings from various studies and identify key summaries regarding perinatal depression in Ethiopian women. Such an initiative will provide crucial insights to inform healthcare practices, guide policy decisions, and ultimately improve maternal and child health outcomes in the region.

Therefore, the primary aim of this umbrella review was to systematically evaluate and consolidate existing evidence on the prevalence of depressive symptoms among perinatal women in Ethiopia. By synthesizing data from multiple systematic reviews and meta-analyses, we aimed to provide a robust overview of the prevalence rates.

## 2. Methods

### 2.1. Reporting and protocol registration

We did this umbrella review of the systematic review and meta-analysis of studies on the prevalence of depressive symptoms among women in the peripartum period (pregnancy and postpartum period). While doing and reporting this umbrella review, the Preferred Reporting Items for Review of Reviews (PRIOR) Checklist has been used as a reference. The steps that are taken in each section of the review to adhere to these guidelines are indicated in S1 File. The protocol of this umbrella review is registered on the International Register of Systematic Reviews (PROSPERO) with registration number: **CRD42023495174**.

### 2.2. Eligibility criteria

In this umbrella review, we included systematic review and meta-analysis studies that fulfill the following pre-defined inclusion criteria based on the Population, intervention, comparator and outcome (PICO) parameter; [1] reported data on the prevalence of perinatal depressive symptoms (i.e., antenatal, postnatal, or the whole perinatal depressive symptoms); [2] studied on women of the perinatal period; and [3] Published in English language. Studies solely on the determinant factors of peripartum depression, non-systematic review/meta-analysis, conducted on non-perinatal women, studies done on populations other than women, studies done in the context outside Ethiopia, and non-peer-reviewed articles that do not provide data on the prevalence of perinatal depression, were excluded from the umbrella review (Table 1).

**Table 1:** Articles were excluded from this umbrella review if they met one or more of the following exclusion criteria.

Exclusion criteria	Explanation
Not perinatal women	The study excluded articles on women who were neither in the perinatal nor the antenatal period
The outcome is not depressive disorder	Studies on perinatal women that did not include perinatal depression as an outcome variable were excluded.
Study context not in Ethiopia	The review also excluded studies on peripartum depression outside the context of Ethiopia
Wrong study type	Non-systematic review/meta-analysis, and non-peer-reviewed articles, studies on non-pregnant women, letters to the editor, opinion pieces, and newsletters were also excluded
Duplicated studies	Duplicate studies identified in the EndNote reference manager were excluded
Grey literature	Non-published articles in repositories
Studies done solely on associated factors	Review studies only assessing the associated factors for perinatal depression
Studies conducted elsewhere	Studies conducted on perinatal women in countries outside of Ethiopia
Not published in English	Studies that are published in a non-English language

### 2.3. Data sources and searches

We searched three bibliographic databases, including PubMed, EMBASE, and PsycINFO, from the inception to 2024. An initial search was done on October 29, 2023, and a search update was again done on September 17, 2024. The search strategy was first developed for the PubMed databases using the following keywords and MeSH terms: Perinatal period, antenatal period, postnatal period, depression, depression symptoms, systematic review and meta-analysis, and Ethiopia. It was adapted to search in the remaining databases (S2 File). The reference lists of umbrella reviews conducted in low- and middle-income countries and of systematic reviews conducted in Ethiopia were also checked to identify systematic reviews and meta-analyses on perinatal depression in Ethiopia. Finally, a consultation of experts in perinatal mental health was also done to identify additional articles.

### 2.4. Data extraction

In this umbrella review, two of the authors (MN and NB) did the data extraction independently using a pre-designed data extraction template. Any discrepancies in the quality measure of the included articles between these two authors were reconciled with a discussion with a third author (MT). Sometimes during the process, authors of the included studies were consulted to ascertain missing data. We extracted all the important data using the following data extraction template. The first author’s name, year of publication, the number of primary studies included, and the specific population studied (antenatal women, postnatal women, and women of the perinatal period). We also extracted data regarding the total sample size included in the review, the total number of cases (number of perinatal women having antenatal, postnatal, or overall perinatal depressive symptoms), and the reported pooled prevalence of depressive symptoms in the systematic review and meta-analysis studies (pooled antenatal, postnatal, or perinatal depressive symptoms). A sample of the data extraction template is attached in the supplementary materials (S3 File).

### 2.5. Overlap of studies between systematic review and or meta-analysis

As many primary articles were included in more than one systematic review and meta-analysis study, there was a need to estimate and report the degree of overlap of such primary studies in the included systematic review and meta-analysis studies. As recommended by Pieper et al., [34] we used the novel graphic approach [35] and estimated the corrected covered area (CCA) to know the degree of overlap. CCA depicts a quantitative measure of the degree of overlap of primary studies between systematic review and meta-analysis studies. A CCA of < 5%, > 5 and ≤ 10%, > 10 and ≤ 15%, and > 15% was interpreted as slight overlap, moderate overlap, high overlap, and very high overlap, respectively [34].

### 2.6. Study quality

A measurement tool to assess systematic reviews (AMSTAR) [36] has been used as an appraisal tool to assess the quality of included systematic reviews and meta-analytic studies. The AMSTAR is a quality assessment tool for evaluating the quality of included systematic reviews and meta-analytic studies in the umbrella review. The AMSTAR tool consisted of the following eleven parameter questions: Was a priori design provided? Was there duplicate study selection and data extraction? Was a comprehensive literature search performed? Was the status of publication (i.e., grey literature) used as an inclusion criterion? Was a list of studies (included and excluded) provided? Were the characteristics of the included studies provided? Was the scientific quality of the included studies assessed and documented? Was the scientific quality of the included studies used appropriately in formulating conclusions? 9. Were the methods used to combine the findings of studies appropriate? Was the likelihood of publication bias assessed? And was the conflict of interest included? Two members of the research team (MN and SY) independently evaluated the quality of eight included systematic reviews and meta-analyses using the 11 parameters of the AMSTAR tool. Any variation between them during scoring was solved with a discussion with the third researcher (AB). Finally, scores were categorised into three categories: scores of 8–11, 4–7, and 0–3 as High, Medium, and Low quality, respectively.

### 2.7. Data synthesis and analysis

All analyses were performed with Stata 16.1. The random-effect meta-analysis was done to estimate the pooled prevalence of peripartum depressive symptoms. We have estimated the heterogeneity among included studies with Higgs I^2^ [37].

Subgroup and sensitivity analyses [38–40] were carried out to explore potential sources of heterogeneity. We did a subgroup analysis of perinatal depressive symptoms by the period (antepartum depressive symptoms and postpartum depressive symptoms). Meta-XL version 5.3 [41] and STATA14 meta-prop packages [42] were used in the analysis.

We have used a p-value of less than 0.05 as a statistically significant criterion during the analysis process.

## 3. Results

### 3.1. Selection of studies

**Fig 1:**
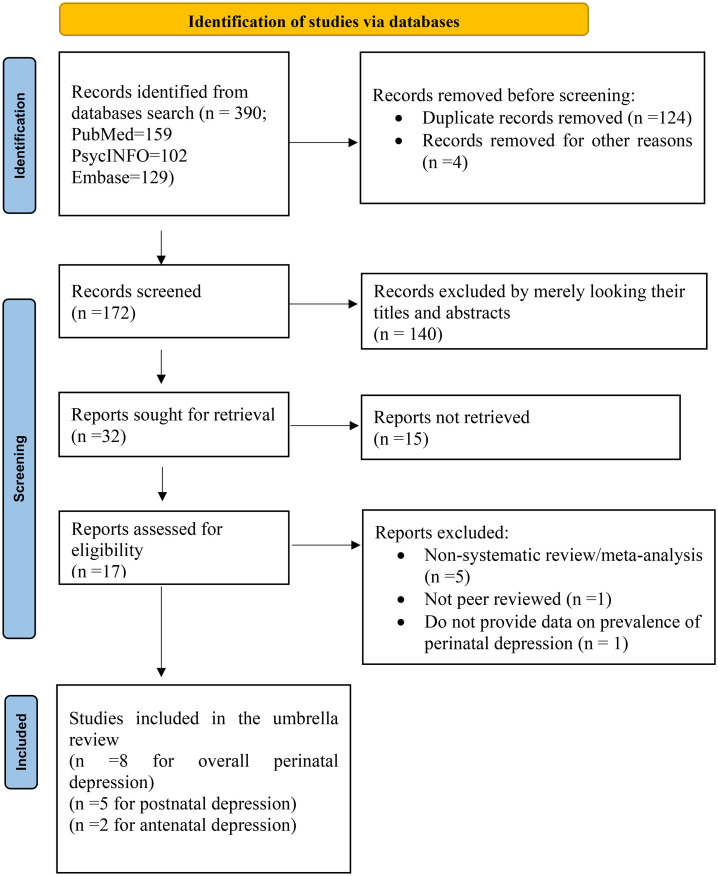
PRISMA flow diagram.

Our extensive search of available literature resulted in 390 papers. Of these records, 124 were excluded since they were duplicates, and a further 140 articles were excluded by reading their titles and abstracts. Seventeen research articles were fully assessed for eligibility. However, only 8 studies were included in the overall umbrella review analysis for perinatal depressive symptoms, and five and two were included in the umbrella review analysis for postnatal and antenatal depression, respectively. The remaining seven articles were excluded due to a variety of reasons (5 were not systematic reviews/meta-analyses, 1 was not peer-reviewed, and 1 did not provide data on the prevalence of perinatal depression) (Fig 1**).**

### 3.2. Characteristics of included studies

This umbrella review investigated perinatal (antenatal or postnatal) depression among eight systematic review and meta-analysis studies [1,6,9, 18–21,30]. Five of the systematic review and meta-analysis studies [6,18,20,21,30] assessed depression among 15 primary studies and 10,540 participants during the postnatal period while two studies [1,9] assessed depression in 8 primary studies and 3859 participants during pregnancy. The remaining systematic review study [19] reported both antenatal and postnatal depression in five unique primary studies and 1193 participants. Overall, a total of 28 unique primary studies with 15,592 participants were included. Systematic review and meta-analysis studies included in this umbrella review were published between 2018 [9,19] and 2021 [18,30] (Table 2**).**

**Table 2:** The characteristics of studies included in this umbrella review (N = 8).

Author Year	Databases covered in the search	Number of primary studies	Sample Size	Cases	Prevalence of Peri-natal depression	
					Prevalence of antepartum depression	Prevalence of postpartum depression
Tolossa et al.2020	Medline, PubMed, Cochrane Library, the Web of Science, Google Scholar, and Scopus	12	9674	2102		22.89%
Zeleke TA et al.2021	MEDLINE, PubMed, PsycINFO, Web of Science, EMBASE, CINAHL, Scopus, and The Cochrane Library	11	7582	1427		22.08%
Mersha, AG, et al.2018	MEDLINE, Scopus, PubMed, Science Direct, and Google Scholar	8	4624	1193	25.8%	
Necho et al.2021	PubMed, Scopus, and EMBASE	16	11,400	2243		21.9%
Ayano G et al.2019.	PubMed, EMBASE, and SCOPUS	5	2126	453	21.28%	
Zegeye et al.2018	PubMed, Google Scholar, Science Direct, and Cochrane Library	10	4983	1206	24.2%	
M.Desta et al.2020	PubMed, Web of Science, SCOPUS, CINAHL, PsycINFO, Google Scholar, Science Direct, and the Cochrane Library	13	9084	1958		21.55%
Duko et al.2020	PubMed, SCOPUS, EMBASE, and Google Scholar	5	4751	955		20.1%

### 3.3. Overlap of studies between systematic review and or meta-analysis

This umbrella review of systematic review and meta-analysis studies included 28 unique primary studies. Of these, seven were included in one systematic review and meta-analysis study, another seven were cited in two SR and MA studies, five were cited in three SR and MA studies, three were cited in four SR and MA studies six were cited in 5 SR and MA studies. The total corrected covered area was 25.5% implying a very high degree of overlap. Details of the overlap between included systematic review and meta-analysis studies are illustrated in Table 3.

**Table 3:** Graphical representation of the degree of overlap of Primary Studies in Systematic Review studies included in Umbrella Reviews.

Primary studies	Systematic review and meta-analysis studies							
Study ID	Tolossa et al.2020	Zeleke TA et al.2021	Mersha,AG, et al.2018	Necho et al.2021	Ayano G et al.2019.	Zegeye et al.2018	M.Desta et al.2020	Duko et al.2020
Kerie et al.2018	X	X		X			X	X
Abadiga 2019	X	X		X			X	
Toru et al.2018	X	X		X			X	X
Abebe Tesfaw et al.2019	X	X		X		X	X	
Asaye Muche et al.2020		X					X	
Shitu Geda et al.2019	X	X		X			X	
Shewangizaw Tadesse et al.2018	X	X		X			X	X
Fantahun Cherie et al.2018	X	X		X			X	X
Adamu and Adinew et al.2018	X						X	
Wubetu, engidaw et al.2020				X			X	
Azale, Fekadu et al.2019	X	X		X			X	X
Bitew, Hanlon et al.2019			X		X	X	X	
Anato, Baye, et al.2019				X			X	
Bisetegn TA et al.2016					X	X		
Ayele TA et al.2016			X		X	X		
Biratu A. et al.2015			X		X	X		
Dibaba Y et al.2015			X		X	X		
Mossie TB et al.2015			X		X	X		
Mariam et al.2016		X	X	X				
Teferra benti et al.2015			X	X				
JN baumgartner et al.2014				X				
Bekele et al. 2017			X					
Teshome H et al.2016	X	X						
Sahile et al.2017						X		
Asmeret Andebirhan 2015						X		
Gemeta WA et al. 2014						X		
Ashish K et al. 2019	X							
Deribachew H et al.2016	X							

**Key**: Corrected covered area was calculated as = (N-r)/ (rc-r) =25.5% where,

c = The number of included systematic review studies (number of columns) =8

r = The number of publications of primary studies (number of tows) =28

N = Number of total primary studies included in the systematic reviews, including double counting/ number of “X” = 78

### 3.3. Quality assessment findings

The AMSTAR quality scoring guide has been used to identify studies with low, medium, and high methodological quality. Accordingly, fifty percent (four of the systematic review/meta-analysis studies) hit the high-quality range, whereas the remaining four rely on the medium-quality range. Only two of the eight included systematic review/meta-analysis studies reported a protocol registration number, and none of the studies reported the excluded studies during analysis (S4 File**).**

### 3.4. Pooled umbrella prevalence of Perinatal depressive symptoms

We included only 28 unique primary studies in this meta-analysis, excluding duplicates to minimise the risk of bias. The prevalence of perinatal depressive symptoms among the included systematic review and meta-analysis studies ranges from 20.1% to 25.8% [1,6,9, 18-21,30].

**Fig 2:**
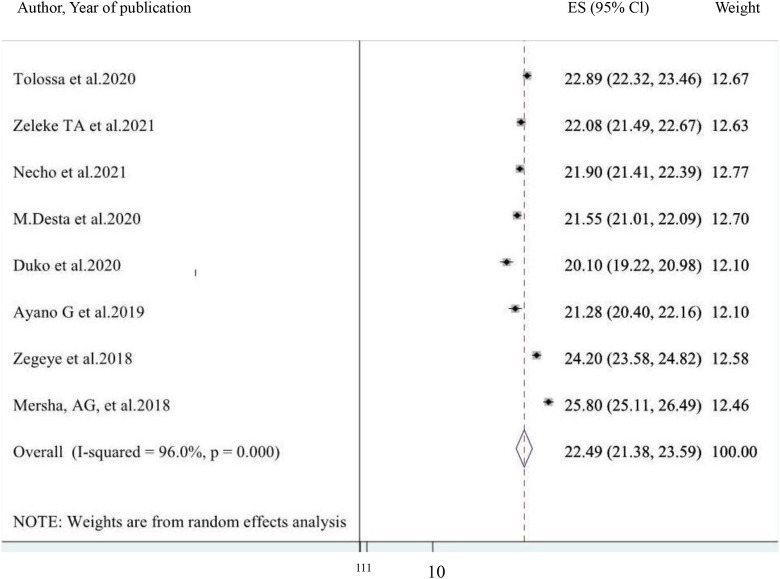
A forest plot for the pooled prevalence of perinatal depression in Ethiopia.

The overall pooled umbrella prevalence of perinatal depressive symptoms across eight included studies in Ethiopia was 22.49% (95% CI: 21.38, 23.59) (Fig 2**),** with significant heterogeneity among studies (I2 = 96.0%, p = 0.000). This pooled prevalence of perinatal depressive symptoms was derived from eight systematic reviews and meta-analyses, encompassing 28 primary studies.

### 3.5. Subgroup analysis by perinatal period (Antenatal period vs Postnatal period)

**Fig 3:**
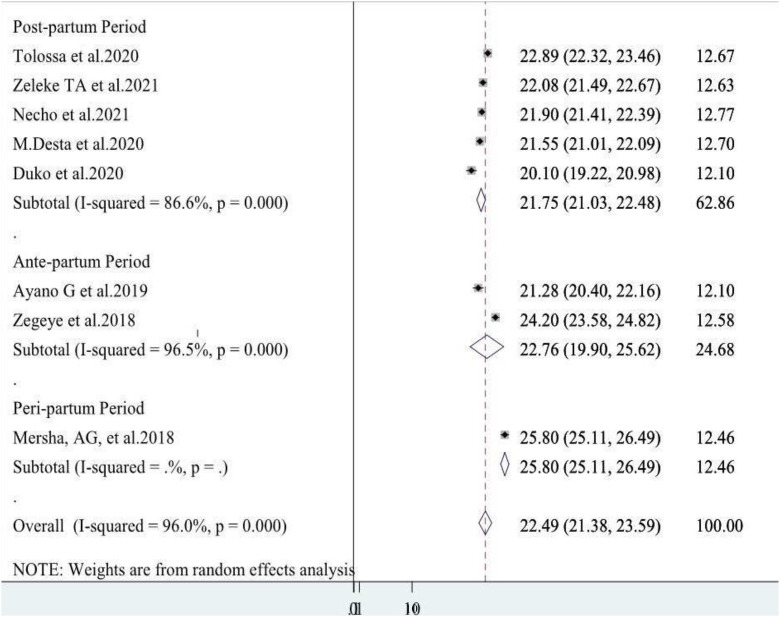
A forest plot for the subgroup analysis of perinatal depression in Ethiopia.

Two systematic review and meta-analysis studies reported ante-natal depressive symptoms and pooled umbrella prevalence estimate was 22.76% (96%CI: 19.90, 25.62) (I^2^ = 96.0%, p = 0.000) whereas five systematic review and meta-analysis studies reported data on postnatal depressive symptoms and the pooled umbrella prevalence estimate was 21.75% (96%CI: 21.03, 22.48) (I^2^ = 86.60%, p = 0.000**) (**Fig 3**).**

### 3.6. Sub-group analysis by the number of studies included in the systematic review and meta-analysis

A subgroup analysis had also been done based on the number of included studies in the included systematic review and meta-analysis studies. Four systematic review and meta-analysis studies included above 10 primary studies and the pooled umbrella prevalence of peripartum depression in these four systematic review and meta-analysis studies was 22.10%;95%CI: 21.55, 22.65; I^2^ = 75.1% and the pooled umbrella prevalence of peripartum depression in studies that included 10 or below primary studies was 22.86%;95%CI: 20.39, 25.33; I^2^ = 97.7% **(See** S5 File).

### 3.7. Sensitivity analysis

We did a one-study leave-out at a time sensitivity analysis to further investigate the source of heterogeneity. Consequently, the result of the analysis showed that the pooled estimate of perinatal depression found when each of the eight included studies is left out from the analysis at a time is within the 95% confidence interval of the pooled estimate obtained when all eight studies are included. This implies that no single influential study outweighed the result of the current umbrella review on the pooled perinatal depression (S6 File: Sensitivity analysis for the prevalence of perinatal depression in Ethiopia).

## 4. Discussion

Umbrella reviews are the high-level synthesis of the evidence and address research questions broader in scope than those examined in individual SR & MA [43]. An umbrella review presents the most comprehensive and robust synthesis of scientific evidence to inform decision-making [44]. Even though many cross-sectional and systematic review-meta-analysis studies were done on perinatal depressive symptoms, to the knowledge of our knowledge, this is the first umbrella review that provides a robust synthesis of the prevalence of perinatal depressive symptoms in Ethiopia. The current umbrella review incorporated eight systematic reviews and meta-analysis studies and 28 primary articles with 15,592 participants. Five of the systematic review and meta-analysis studies [6,18,20,21,30] assessed depressive symptoms among 15 primary studies and 10,540 participants during the postpartum period, while the remaining two studies [1,9] assessed depressive symptoms in 8 primary studies and 3859 participants during pregnancy.

The current umbrella review highlighted that there is a high prevalence of perinatal depression symptoms in Ethiopia, 22.49% and ranging from 21.38% to 23.59%**.** The pooled prevalence of depressive symptoms during the antenatal period and postnatal period was also found to be 22.76% (varying from 19.90% to 25.62%) and 21.75% (ranging from 21.03% to 22.48%), respectively.

This was lower as compared to the pooled global prevalence of antepartum depression from an umbrella review study during the COVID-19 pandemic [45]. The study gathered data from five continents and 45 countries, and the pooled prevalence of antenatal depression was 29%. Cultural differences [46], COVID-19-related variables like restrictions imposed to limit the contagion rates and perceived severity of COVID-19 might be attributed to the higher pooled prevalence during this period.

This umbrella review reported higher prevalence rates of perinatal depression as compared to high-income countries, where the reported estimated perinatal depression generally ranges from 10% to 17% [47,48]. Possible contributing factors to these higher prevalence rates in Ethiopia may include non-robust healthcare systems, poor access to mental health services, and relatively poor awareness of society of mental health issues [26,49,50]. Additionally, Ethiopia’s healthcare system faces challenges such as limited resources, insufficient training for healthcare providers, and cultural stigma surrounding mental health, all of which contribute to the higher prevalence observed [26,51–53]. In contrast, the availability of early interventions, comprehensive prenatal care, and postnatal support in high-income countries may mitigate the risk of developing perinatal depression.

In comparison to the findings of the current umbrella review, reviews in middle-income countries present a mixed picture with pooled prevalence estimates of perinatal depression falling between 15% to 25% [10,54]. This means that reported pooled rates of perinatal depression in middle-income countries showed both higher and lower prevalence estimates, as compared with the findings from the current umbrella review. This variability can be attributed to differing socio-economic conditions, healthcare access, and cultural attitudes toward perinatal mental health [10,55,56]. In many middle-income nations, rapid urbanization and economic shifts may exacerbate stressors for pregnant and postpartum women, leading to increased rates of depression [57–59]. Ethiopia, as a low-middle-income country, shares similarities with these regions, highlighting the need for targeted interventions that consider both economic factors and cultural contexts.

The pooled prevalence of perinatal depression in the current study often mirrors that of prevalence estimates in low-income countries, with rates ranging from 20% to 30% [60–62]. These high prevalence rates both in Ethiopia and other low-income countries could frequently be linked to pervasive socio-economic challenges, including poverty, gender inequality, and lack of access to quality healthcare [63–67]. Similar to Ethiopia, many low-income settings struggle with inadequate mental health services and a lack of trained professionals to address the mental health needs of women during the perinatal period. The parallels between Ethiopia and other low-income countries indicate a shared need for comprehensive approaches that not only enhance mental health support but also address the underlying socioeconomic determinants of health.

This umbrella review of systematic reviews and meta-analytic studies on perinatal depression has the following strengths. [1]: It is the first umbrella review to quantify the pooled prevalence of perinatal depressive symptoms in Ethiopia. [2]: the quantification and interpretation of the degree of overlap of primary studies between the included systematic review and meta-analysis studies using a corrected coverage area (CCA). [3] The determination and interpretation of the quality of included systematic reviews and meta-analysis studies using a stringent AMSTAR tool. [4] The estimation of the pooled prevalence of depressive symptoms at different periods (antepartum period & postnatal period). This umbrella review, however, has the following limitations. Firstly, the primary studies in the included systematic review and meta-studies vary in methodological rigor, sample sizes, and measurement tools, which may affect the reliability of the pooled prevalence estimate. Secondly, all systematic review and meta-analysis studies included in the umbrella review were synthesized from cross-sectional primary studies, limiting the ability of this review to draw causal inferences.

A significant degree of overlap among primary studies could result in the overestimation of perinatal depressive symptoms, affect the precision of findings, and potentially lead to a biased conclusion of the study. Therefore, such limitations should be considered when using the study findings from this umbrella review. The presence of a high degree of heterogeneity among included studies should also be considered in the interpretation of its findings.

## 5. Conclusion

This umbrella review highlights a concerning pooled prevalence of perinatal depression in Ethiopia, estimated at 22.49% (22.76% for antenatal depression symptoms and 21.75% for postnatal depressive symptoms). This figure underscores the critical need for heightened awareness and targeted interventions in maternal mental health services across the country. Given the multifaceted nature of perinatal depression and its implications for both maternal and child health, it is essential to implement comprehensive screening and support programs tailored to the cultural and socioeconomic contexts of Ethiopian women.

The findings also point to significant gaps in the existing literature, suggesting a need for more robust and diverse studies to better understand the risk factors and barriers to care. Addressing perinatal depression requires a multi-disciplinary approach that includes healthcare providers, policymakers, and community stakeholders. By prioritizing mental health in maternal care, we can improve outcomes for mothers and their children, ultimately contributing to healthier families and communities in Ethiopia. Further research is imperative to develop effective interventions and inform public health strategies aimed at reducing the burden of perinatal depression in this population.

## 6. Implications from the findings of this umbrella review of perinatal depression in Ethiopia

The pooled prevalence of perinatal depression symptoms in Ethiopia, estimated at 22.49%, highlights a significant public health concern that necessitates urgent attention and action. This high pooled prevalence indicates that approximately one in five women may experience depressive symptoms during the perinatal period, which can have profound implications for maternal and child health.

The findings underscore the need for healthcare systems in Ethiopia to integrate mental health screening and support into routine prenatal and postnatal care. Training healthcare providers to recognize and address perinatal depression can enhance maternal well-being and improve outcomes for infants.

Policymakers should also prioritize mental health in maternal health policies, ensuring that resources are allocated for mental health services. This includes funding for community-based interventions and support programs that target vulnerable populations. Increased awareness campaigns are crucial to destigmatize mental health issues related to perinatal depression. Educational initiatives for women, families, and communities can help promote understanding of the condition and encourage women to seek help.

There is also a pressing need for further research to identify risk factors specific to the Ethiopian context, as well as the effectiveness of various intervention strategies. Longitudinal studies could provide insights into the long-term effects of perinatal depression on maternal and child health.

In general, addressing perinatal depression effectively requires collaboration across sectors, including health, education, and social services. A multisectoral approach can facilitate comprehensive care that addresses the social determinants of health contributing to mental health challenges.

By recognizing the substantial burden of perinatal depression in Ethiopia, stakeholders can work collaboratively to implement effective strategies aimed at prevention, early detection, and management, ultimately improving the health and well-being of mothers and their children.

## Supporting information

S1 File A PRIOR Checklist for this umbrella review of systematic review and meta-analysis studies. (DOCX)

S2 File Search strategy for this umbrella review of systematic review and meta-analysis studies. (DOCX)

S3 File Data extraction template for the Prevalence of Perinatal depression in Ethiopia: An Umbrella Review of Systematic Review and Meta-analysis Studies. (DOCX)

S4 File AMSTAR score of included systematic review and meta-analysis studies for the umbrella review on depressive symptoms among peripartum (Antepartum and postpartum) women in Ethiopia. (DOCX)

S5 File A forest plot for the subgroup analysis of the prevalence of peripartum depression based on the number of included studies. (DOCX)

S6 File Sensitivity analysis for the prevalence of perinatal depression in Ethiopia. (DOCX)
