# Supplementary material for: Prevalence of perinatal depression in Ethiopia: An umbrella review of systematic review and meta-analysis studies
Source: PLoS One. 2026 Apr 27;21(4):e0347570. doi: 10.1371/journal.pone.0347570 (PMC13120232; doi:10.1371/journal.pone.0347570)
Supplement: S2 File — (DOCX) [file pone.0347570.s002.docx]

**Supplementary File 2**

1. **Search strategy for this umbrella review of systematic review and meta-analysis studies**

| **Search Engine** | **Search terms** | **Result** |
| --- | --- | --- |
| **Pubmed** | **(Perinatal period) OR (antenatal period) OR (postnatal period) AND (depression) OR (depression symptoms) AND (*systematic review and meta-analysis *) AND (Ethiopia)** | **159** |
| **PsycINFO** | **(Perinatal period) OR (antenatal period) OR (postnatal period) AND (depression) OR (depression symptoms) AND (*systematic review and meta-analysis *)) AND (Ethiopia)** | **102** |
| **Embase** | **(Perinatal period) OR (antenatal period) OR (postnatal period) AND (depression) OR (depression symptoms) AND (*systematic review and meta-analysis *)) AND (Ethiopia).** | **129** |

1. **Sample of the excluded studies during the search process for eligible studies in this umbrella review**

| **Publication year** | **Citation** | **Reason for exclusion** |
| --- | --- | --- |
| **2024** | [**Exploring paternal postpartum depression and contributing factors in Ethiopia: a systematic review and meta-analysis.**](https://pubmed.ncbi.nlm.nih.gov/39478469/)  **Kitil GW, Hussen MA, Chibsa SE, Chereka AA** | **Conducted in a non-target population** |
| **2023** | [**Poor sleep quality and suicidal ideation among pregnant women during COVID-19 in Ethiopia: systematic review and meta-analysis.**](https://pubmed.ncbi.nlm.nih.gov/37790617/)  **Hasen AA, Seid AA, Mohammed AA.** | **Assessed a non-desired outcome for the study** |
| **2021** | **P**[**revalence and determinants of depression among old age: a systematic review and meta-analysis.**](https://pubmed.ncbi.nlm.nih.gov/34922595/)  **Zenebe Y, Akele B, W/Selassie M, Necho M.** | **Difference in target population and context of the study** |
| **2021** | [**The prevalence of depression among adolescent with HIV/AIDS: a systematic review and meta-analysis.**](https://pubmed.ncbi.nlm.nih.gov/33906698/)  **Ayano G, Demelash S, Abraha M, Tsegay L.** | **Difference in target population and context of the study** |
| **2021** | [**The relationship between social support and mental health problems during pregnancy: a systematic review and meta-analysis.**](https://pubmed.ncbi.nlm.nih.gov/34321040/)  **Bedaso A, Adams J, Peng W, Sibbritt D** | **Conducted out of the context of the study** |
| **2020** | [**Epidemiology of antenatal depression in Africa: a systematic review and meta-analysis.**](https://pubmed.ncbi.nlm.nih.gov/32345263/)  **Dadi AF, Wolde HF, Baraki AG, Akalu TY** | **Conducted out of the context of the study** |
| **2023** | [Global prevalence of post abortion **depression**: **systematic** **review** and **Meta**-**analysis**.](https://pubmed.ncbi.nlm.nih.gov/37884930/)  Gebeyehu NA, Tegegne KD, Abebe K, Asefa Y, Assfaw BB, Adella GA, Alemu BW, Sewyew DA | **Conducted in a non-target population and out of the context** |
| **2023** | [**Depression and determinants among diabetes mellitus patients in Ethiopia, a systematic review and meta-analysis.**](https://pubmed.ncbi.nlm.nih.gov/36991387/)  **Tegegne KD, Gebeyehu NA, Kassaw MW** | **Conducted in a non-target population** |
| **2024** | [**Systematic review and meta-analysis on the prevalence and associated factors of depression among hypertensive patients in Ethiopia.**](https://pubmed.ncbi.nlm.nih.gov/38917087/)  **Tassew WC, Nigate GK, Assefa GW, Zeleke AM, Ferede YA** | **Conducted in a non-target population** |
| **2024** | [**Anxiety and depression among cancer patients in Ethiopia: a systematic review and meta-analysis.**](https://pubmed.ncbi.nlm.nih.gov/38455516/)  **Geremew H, Abdisa S, Mazengia EM, Tilahun WM, Haimanot AB, Tesfie TK, Mneneh AL, Mengie MG, Endalew B, Birhanu MY, Asmare L, Simegn MB** | **Conducted in a non-target population** |
| **2022** | [**Prevalence and factors associated with depression among older adults in the case of a low-income country, Ethiopia: a systematic review and meta-analysis.**](https://pubmed.ncbi.nlm.nih.gov/36320004/)  **Kasa AS, Lee SC, Chang HR** | **Conducted in a non-target population** |
| **2024** | [**Prevalence of depression and its associated factors among Ethiopian students: a systematic review and meta-analysis.**](https://pubmed.ncbi.nlm.nih.gov/38834322/)  **Fentahun S, Takelle GM, Rtbey G, Andualem F, Tinsae T, Nakie G, Melkam M, Tadesse G** | **Conducted in a non-target population** |
| **2021** | [**Prevalence of depression among women with obstetric fistula in low-income African countries: a systematic review and meta-analysis.**](https://pubmed.ncbi.nlm.nih.gov/32221701/)  **Duko B, Wolka S, Seyoum M, Tantu T.** | **Difference in the target population and context of the study** |
