# Supplementary material for: Prevalence of perinatal depression in Ethiopia: An umbrella review of systematic review and meta-analysis studies
Source: PLoS One. 2026 Apr 27;21(4):e0347570. doi: 10.1371/journal.pone.0347570 (PMC13120232; doi:10.1371/journal.pone.0347570)
Supplement: S3 File — (DOCX) [file pone.0347570.s003.docx]

**Supplementary File 3:** Data extraction template for the Prevalence of Perinatal depression in Ethiopia: An Umbrella Review of Systematic Review and Meta-analysis Studies

| S. No | Names of authors of the SR and MA study | Year of publication | Databases covered in the SR &MA | Number of primary studies included in the SR & MA | Population description (Antenatal, postnatal, or perinatal women) | Total samples included in the SR & MA | Number of cases of perinatal depression | Pooled prevalence of perinatal depression | Pooled prevalence of antenatal depression | Pooled prevalence of postnatal depression |
| --- | --- | --- | --- | --- | --- | --- | --- | --- | --- | --- |
|  |  |  |  |  |  |  |  |  |  |  |
|  |  |  |  |  |  |  |  |  |  |  |
|  |  |  |  |  |  |  |  |  |  |  |
|  |  |  |  |  |  |  |  |  |  |  |
