# Supplementary material for: Prevalence of perinatal depression in Ethiopia: An umbrella review of systematic review and meta-analysis studies
Source: PLoS One. 2026 Apr 27;21(4):e0347570. doi: 10.1371/journal.pone.0347570 (PMC13120232; doi:10.1371/journal.pone.0347570)
Supplement: S4 File — (DOCX) [file pone.0347570.s004.docx]

**Supplementary File 4:** AMSTAR score of included systematic review and meta-analysis studies for the umbrella review on depressive symptoms among peripartum (Antepartum and postpartum) women in Ethiopia.

| AMSTAR criteria | Name of the systematic review and meta-analysis | | | | | | | |
| --- | --- | --- | --- | --- | --- | --- | --- | --- |
|  | Tolossa et al.2020 | Zeleke TA et al.2021 | Mersha, AG, et al.2018 | Necho et al.2021 | Ayano G et al.2019. | Zegeye et al.2018 | M.Desta et al.2020 | Duko et al.2020 |
| 1. Was a priori design provided? | **X** | **✓** | **X** | **X** | **X** | **X** | **X** | **✓** |
| 2. Was there duplicate study selection and data extraction? | **✓** | **✓** | **✓** | **✓** | **✓** | **✓** | **✓** | **✓** |
| 3. Was a comprehensive literature search performed? | **✓** | **✓** | **✓** | **X** | **X** | **✓** | **✓** | **X** |
| 4. Was the status of the publication (e.g. grey literature) used as inclusion criteria? | **✓** | **X** | **X** | **X** | **✓** | **✓** | **X** | **X** |
| 5. Was a list of included and excluded studies provided? | **X** | **X** | **X** | **X** | **X** | **X** | **X** | **X** |
| 6. Were the characteristics of included studies provided? | **✓** | **✓** | **✓** | **✓** | **✓** | **✓** | **✓** | **✓** |
| 7. Was the scientific quality of the included studies assessed and reported? | **✓** | **✓** | **✓** | **✓** | **✓** | **X** | **✓** | **✓** |
| 8. Was the scientific quality of included studies used appropriately in formulating conclusions? | **X** | **✓** | **X** | **✓** | **✓** | **X** | **✓** | **✓** |
| 9. Were the methods used to combine the findings of the study appropriate? | **✓** | **X** | **✓** | **✓** | **✓** | **✓** | **✓** | **✓** |
| 10. Was the likelihood of publication bias assessed? | **X** | **X** | **✓** | **✓** | **✓** | **✓** | **✓** | **✓** |
| 11. Was the conflict of interest stated? | **✓** | **✓** | **✓** | **✓** | **✓** | **✓** | **✓** | **✓** |
| Total AMSTAR Score Grading | **7** | **7** | **7** | **7** | **8** | **8** | **8** | **8** |
