# Supplementary material for: Prevalence of perinatal depression in Ethiopia: An umbrella review of systematic review and meta-analysis studies
Source: PLoS One. 2026 Apr 27;21(4):e0347570. doi: 10.1371/journal.pone.0347570 (PMC13120232; doi:10.1371/journal.pone.0347570)
Supplement: S5 File — (DOCX) [file pone.0347570.s005.docx]

|   **Supplementary File 5:** A forest plot for the subgroup analysis of the prevalence of peripartum depression based on the number of included studies |
| --- |
