# Supplementary material for: Prevalence of perinatal depression in Ethiopia: An umbrella review of systematic review and meta-analysis studies
Source: PLoS One. 2026 Apr 27;21(4):e0347570. doi: 10.1371/journal.pone.0347570 (PMC13120232; doi:10.1371/journal.pone.0347570)
Supplement: S6 File — (DOCX) [file pone.0347570.s006.docx]

|   **Supplementary File 6:** Sensitivity analysis for the prevalence of perinatal depression in Ethiopia |
| --- |
